# Kinotypes: stable species- and individual-specific profiles of cellular kinase activity

**DOI:** 10.1186/1471-2164-14-854

**Published:** 2013-12-05

**Authors:** Brett Trost, Jason Kindrachuk, Erin Scruten, Philip Griebel, Anthony Kusalik, Scott Napper

**Affiliations:** 1 Department of Computer Science, University of Saskatchewan, Saskatoon, Canada; 2 National Institute of Allergy and Infectious Diseases, National Institutes of Health, Emerging Viral Pathogens Section, Frederick, USA; 3 Vaccine and Infectious Disease Organization, University of Saskatchewan, Saskatoon, Canada; 4 School of Public Health, University of Saskatchewan, Saskatoon, Canada; 5 Department of Biochemistry, University of Saskatchewan, Saskatoon, Canada

## Abstract

**Background:**

Recently, questions have been raised regarding the ability of animal models to recapitulate human disease at the molecular level. It has also been demonstrated that cellular kinases, individually or as a collective unit (the kinome), play critical roles in regulating complex biology. Despite the intimate relationship between kinases and health, little is known about the variability, consistency and stability of kinome profiles across species and individuals.

**Results:**

As a preliminary investigation of the existence of species- and individual-specific kinotypes (kinome signatures), peptide arrays were employed for the analysis of peripheral blood mononuclear cells collected weekly from human and porcine subjects (*n* = 6) over a one month period. The data revealed strong evidence for species-specific signalling profiles. Both humans and pigs also exhibited evidence for individual-specific kinome profiles that were independent of natural changes in blood cell populations.

**Conclusions:**

Species-specific kinotypes could have applications in disease research by facilitating the selection of appropriate animal models or by revealing a baseline kinomic signature to which treatment-induced profiles could be compared. Similarly, individual-specific kinotypes could have implications in personalized medicine, where the identification of molecular patterns or signatures within the kinome may depend on both the levels of kinome diversity and temporal stability across individuals.

## Background

Efforts to increase our understanding of the mechanisms of human disease from the perspectives of both gross pathology and molecular pathogenesis have relied heavily on the use of animal models that are assumed to mimic those pathological states. Animal models, in particular those involving mice, have been employed extensively in such investigations as well as for identifying novel therapeutics and assessing their efficacy. However, many studies have relied on the similarities in the phenotypic presentation of disease rather than similarities in the underlying molecular mechanisms. Further confounding these investigations has been the assumed cross-species conservation in identities and physicochemical properties of the host molecular machinery. Although murine models have been employed extensively, there has been a relative paucity of therapeutic candidates that have translated into approved use for humans. These observations have resulted in extensive debate regarding the ability of many animal models to faithfully recapitulate human disease and to accurately predict drug efficacy in humans.

Given this, it would seem prudent to re-evaluate the criteria that drive the selection of a particular species as an animal model. Seok and colleagues recently reported that the genomic responses of mice in acute inflammatory disease models correlated poorly with those of human patients [1]. Although the authors recognized that these prior studies may have been hindered by inadequate study designs, a fatal flaw for many investigations can likely be attributed to the assumption of conservation of host responses between mice and humans. In light of these findings, it has been suggested that a practical solution would be to select animal models based on their conservation of molecular responses to those of humans. Further, for diseases in which human clinical studies are not ethical, selection of animal models that best reflect or mimic human molecular responses would provide increased confidence in the selection or testing of therapeutics. This highlights the need for novel approaches to assess the conservation in molecular responses and identify conserved biomarkers between humans and non-human animals used in disease models.

Analyzing the conservation of molecular responses has applications not only in selecting appropriate animal models, but also in biomarker identification. While the identification and characterization of biomarkers related to disease pathology has resulted in their application to guide the diagnosis and treatment of disease [2,3], the clinical value of such biomarkers in enabling effective diagnosis or treatment guidance is dependent upon their sensitivity and specificity, which are often low [4,5]. Historically, biomarkers have typically represented variations in the sequence, expression or modification of a single biomolecule. While such a simple relationship between a molecular characteristic and a phenotype is attractive from conceptual and practical perspectives, it underestimates the complexity associated with many diseases. Although some diseases are attributable to a single gene, these binary diseases represent the “low hanging fruit” of biomarker discovery. Further, in many cases diseases considered to be genetically determined have been found to display variability that must be attributed to other regulatory or phenotypic differences between individuals. Therefore, it seems appropriate to move beyond the “single gene, single disease” paradigm to a more systematic understanding of health and disease. This shift to more direct phenotypic determinants of disease often requires characterizing molecular mechanisms and biomarkers at informative, global levels. Such a systems biology approach requires examination of the dynamic interplay between large collections of biomolecules. A key challenge for the identification of biomarkers for such multi-faceted phenotypes is the development of technologies that effectively reflect these complex interactions in patient-derived samples. Investigations of dynamic patterns of gene and protein expression, through transcriptional and proteomic approaches, have offered insight into a number of disease-associated phenotypes. In cancer, for example, there are a number of biomarkers that contribute to diagnosis, subtype classification, prognosis and treatment outcomes [6]. Similarly, in hepatitis C virus (HCV) infection, patterns of expression of interferon (IFN)-related genes predict IFN treatment efficacy [7]. While these examples highlight the potential to apply global approaches to understand biology and identify biomarkers, there is concern regarding the inability of these approaches to consider post-transcriptional and post-translational regulatory events.

Kinase-mediated phosphorylation is the predominant mechanism for regulation of protein function. Disruption or dysregulation of kinase activity is associated with a number of pathophysiological states, including cancer, inflammation, neurological disorders and diabetes [8]. Thus, there is considerable interest in defining kinase activities, as well as in manipulating them for therapeutic purposes—an objective facilitated by the fact that kinases are highly “druggable” [8]. As a result, kinases represent a top priority of the pharmaceutical industry [9] and are currently the most frequently targeted gene class for cancer therapies, second only to G protein-coupled receptors across all therapeutic areas [10]. Increased appreciation for the intimate link between kinases and health has prompted the development of technologies to characterize the phosphoproteome or kinome [11], including efforts by our group to utilize peptide arrays for low-cost, high-throughput kinomic characterizations [12-16].

With respect to protein phosphorylation, the simplest evidence for the conservation (or lack thereof) of molecular responses among species lies in the content of the kinome. Genome sequencing has revealed that eukaryotes differ greatly in the number of protein kinases encoded by their genomes. For instance, the human genome encodes approximately 518 protein kinases [17]; in contrast, the proteome of *Arabidopsis thaliana* encodes around 1000 protein kinases [18], while S*accharomyces cerevisiae* encodes fewer than 120 [19]. This suggests that kinase-mediated molecular responses may not be well-conserved among species, and that conclusions drawn from the investigation of protein phosphorylation in one species may not be applicable to another species. On the other hand, a previous study using peptide arrays suggested that despite the very different protein kinase complements in various eukaryotes, the substrates phosphorylated by these organisms exhibit substantial similarities [20]. As such, the level of conservation of kinase-mediated host responses in different species has yet to be fully delineated.

In outbred animals, it is common to observe a range of responses to a given stimulus or condition. This diversity likely reflects a combination of genetic, environmental and situational variables. Similar diversity is also apparent within human populations. In our previous investigations of livestock, unique animal-specific patterns of baseline kinome activities were often observed [12,13]. From these animal-specific baselines, conserved yet variable responses to defined stimuli were found, suggesting that phenotypes are represented within unique cellular kinome environments. Given the close relationship between kinases and phenotype, we hypothesized that these unique signaling patterns could be used as biomarkers.

To probe the existence of species- and individual-specific kinotypes, we applied peptide arrays to conduct kinome analysis of human and porcine peripheral blood mononuclear cells (PBMCs). The peptides on the array represent phosphorylation events for which there is perfect sequence conservation between human and pig, making this array equally applicable for investigating either species. For each species, we considered six individuals sampled once per week for four consecutive weeks. The extent of conservation of kinome activity was evaluated through hierarchical clustering analysis, principal component analysis (PCA), and statistical consideration of the data. Across humans and pigs, there was overwhelming evidence for species-specific kinome profiles. The human subjects, who were variable in terms of age, gender, genetics and lifestyle, also provided evidence for individualized, stable kinome profiles. Similarly, a distinctive kinotype was observed among pigs, where potential sources of variability like age, genetics and lifestyle were minimized. The demonstration of species-specific kinotypes may have applications in the selection of animal models for certain diseases, while the existence of stable, individualized kinotypes within members of the same species may have utility in using phosphorylation-associated biomarkers to guide disease diagnosis and treatment.

## Results

### Raw and normalized array data

For each species (human and pig), one sample was taken from each of six individuals for four consecutive weeks, for a total of 48 samples. Peptide arrays were incubated with each sample, and raw phosphorylation intensity data were collected by scanning the arrays and determining the intensity of each spot (the foreground intensity), as well as the intensity of the slide surrounding that spot (the background intensity). Because the stain binds non-specifically to the slide itself, the background intensity was often greater than the foreground intensity; in fact, only 14% of spots from the human arrays and 31% of spots from the porcine arrays had a raw foreground signal greater than the corresponding background signal. There were also differences among subjects from the same species in terms of the number of spots having a foreground signal above background. However, these systemic variations were eliminated once normalization was performed using VSN. Specifically, the average signal intensity (after background subtraction and normalization) among spots from the human arrays was 11.77 compared to 11.81 for the pig arrays, showing that measurements from the different arrays had successfully been brought onto the same scale. The raw and normalized intensity data for all arrays are available as Additional file 1 and Additional file 2, respectively.

In order to evaluate the technical reproducibility of the arrays, individual peptides (297) were printed nine times per array, and a chi-square test was performed for each unique peptide on a given array to determine the variability amongst these technical replicates. Peptides with P-values <0.01 were designated as inconsistently phosphorylated on that array. Over all 48 arrays, an average of 282 peptides yielded technically reproducible signals within an array (range: 268–296), giving strong evidence for the technical reproducibility of the phosphorylation signal. Due to this strong reproducibility, all 297 peptides were used for subsequent analyses.

### Species-specific kinome profiles

Species-specific variations in phosphorylation-mediated signalling were considered as the initial test for the existence of kinotypes. Pigs were selected for comparison because they are often employed in large animal models of human diseases and therapeutic studies due to conserved biological responses and significant genetic similarities [21,22]. PBMCs were used as they are obtained through non-invasive procedures and require minimal manipulation to isolate. Further, demonstrating a kinotype within this diverse and dynamic cell population would offer confidence that individualized patterns of kinase activity would also be observed in more static and homogenous tissues.

**Figure 1:**
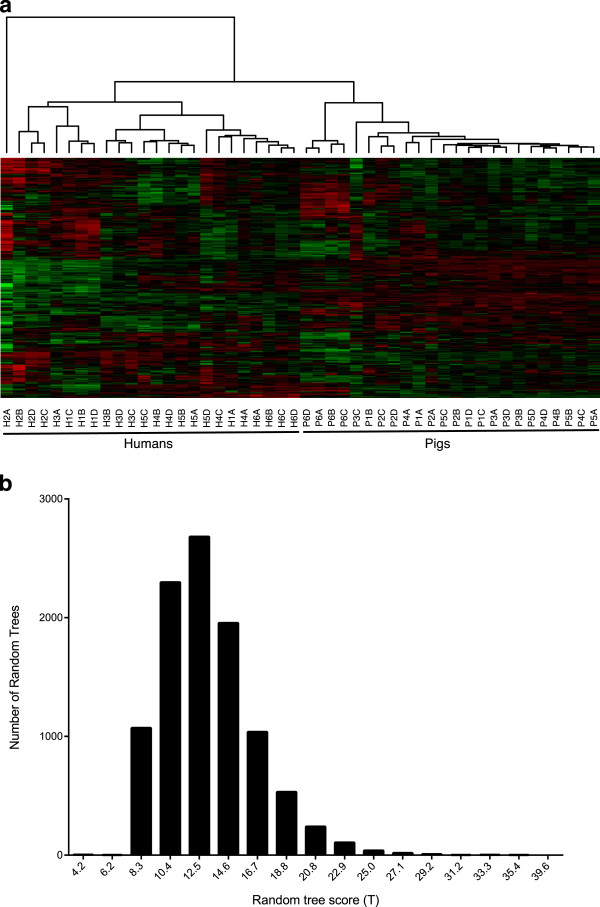
**Clustering of human and porcine kinome profiles. (a)** Hierarchical clustering of human and porcine kinome profiles. The distance metric used was (1 – Pearson correlation), while McQuitty linkage was used as the linkage method. Rows correspond to probes (phosphorylation targets), and columns correspond to samples. The first character of each sample label identifies the species (“H” for human and “P” for pig), the second character identifies the individual from which the sample was taken, and the third indicates the time point. Colors indicate the average (over 9 intra-array replicates) normalized phosphorylation intensity of each target, with red indicating increased phosphorylation and green indicating decreased phosphorylation. The intensity of the color corresponds to the degree of increase or decrease [36]. **(b)** Distribution of random tree scores. The number of random trees having each random tree score is shown. For comparison, the score of the actual tree shown in part A is 97.9.

All human and porcine kinome profiles were analyzed simultaneously using hierarchical clustering. There were significant differences in the profiles of humans and pigs, with nearly perfect species-specific separation of the samples (Figure 1a). Specifically, at the highest level of clustering, the samples separated into sample H2A (the first time point sample of human subject A) and all other samples (perhaps indicating that H2A was an outlier, as all the remaining samples for human subject A clustered exclusively with the other human samples). At the subsequent level, all remaining samples clustered into distinct, species-specific groups. To calculate the extent to which the samples clustered on the basis of species, the scoring metric *T* described in Methods was applied to the binary tree form of the dendrogram. The value of *T* was 97.9 out of 100, indicating near-perfect clustering by species. To determine whether *T* was greater than what would be expected by chance, the score was also calculated for 10,000 random trees. No random tree had a score >39.6 (Figure 1b), giving a P-value <0.0001. This supported the existence of species-specific patterns of kinome activity within human and porcine PBMCs.

### Individual-specific human kinome profiles

**Figure 2:**
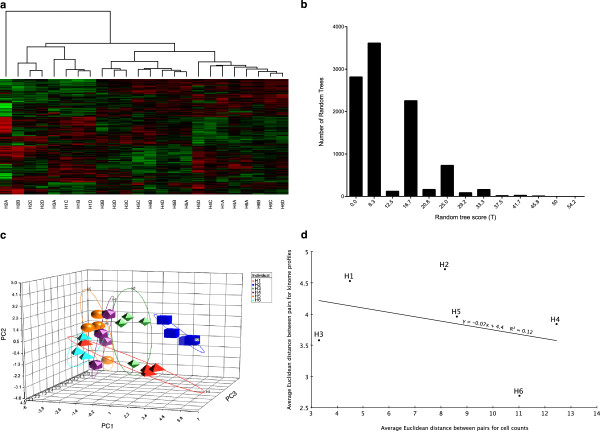
**Clustering of human kinome profiles. (a)** Hierarchical clustering of human kinome profiles. For details, see the caption for Figure 1a. **(b)** Distribution of random tree scores. For comparison, the score of the actual tree shown in part A is 62.5. **(c)** Three dimensional PCA. Individual subjects (H1, H2, H3, H4, H5, and H6) are color-coded. **(d)** Correlation between PBMC composition and kinome profiles. The Euclidean distance was calculated between each of the C (4,2) = 6 possible pairs of samples from the same individual both for cell counts (as given in Table 1) and for kinome profile (average normalized intensity values for each peptide on the corresponding array). The six Euclidean distances were then averaged for a given individual, giving a single number that represents the level of variation in either cell counts or kinome profile for that individual.

Having demonstrated a species-specific kinotype, we investigated whether individual-specific kinomic patterns exist within members of the same species. The human subjects were investigated first as they were considered to be more likely to display significant individual differences due to variability in age, gender, race and lifestyle. Hierarchical clustering analysis revealed a clear trend for samples from the same individual to cluster together (Figure 2a). The score calculated for the corresponding tree was *T =* 62.5. This score was not equaled or exceeded by any of the 10,000 random trees, with the highest random tree score being 54.2, and only 0.6% of the random trees having a score >33.3 (Figure 2b). This comparison again gave a P-value <0.0001, supporting the hypothesis that individual-specific patterns of kinome activity exist within human PBMCs. The results of the clustering analysis were further verified using principal component analysis (PCA). The values of the first three principal components were calculated for each human sample and a three-dimensional scatterplot was created (Figure 2c). As with the hierarchical clustering, there was a strong trend for the kinome profiles to segregate on the basis of individual.

As PBMCs represent a mixed cell population, we assessed whether unique ratios of myeloid or lymphocyte subsets within an individual could be associated with particular signalling patterns. There was minimal variance between individuals with respect to the relative ratio of PBMCs over time (Table 1). The polymononuclear cell counts for the pigs and humans were within the normal ranges of 25-40% and 45-70%, respectively. Furthermore, there was no significant relationship between the white blood cell population variance and signaling profile variance within individuals (Figure 2d).

**Table 1:** Differential white blood cell counts

**Human**			**Pig**		
**ID**	**Lymphocytes**	**Monocytes**	**ID**	**Lymphocytes**	**Monocytes**
H1A	34	10	P1A	55	12
H1B	29	10	P1B	62	1
H1C	35	9	P1C	56	5
H1D	31	6	P1D	78	5
H2A	36	7	P2A	52	8
H2B	37	9	P2B	41	10
H2C	35	2	P2C	64	1
H2D	25	5	P2D	81	8
H3A	59	3	P3A	61	4
H3B	55	5	P3B	68	3
H3C	57	4	P3C	54	5
H3D	54	6	P3D	71	2
H4A	31	7	P4A	50	7
H4B	16	6	P4B	56	5
H4C	40	4	P4C	68	4
H4D	31	5	P4D	65	4
H5A	35	7	P5A	59	7
H5B	37	6	P5B	55	4
H5C	26	8	P5C	67	7
H5D	23	8	P5D	88	5
H6A	27	8	P6A	60	4
H6B	30	6	P6B	69	0
H6C	46	5	P6C	63	3
H6D	38	6	P6D	64	5

The first character in the sample ID column is either “H” for human or “P” for pig. The second character (e.g. “2” in “H2A”) identifies the individual from which the sample was taken, while the third character (e.g. “A”) indicates the time point.

Previously, we demonstrated that monocytes purified from different animals have distinct signaling profiles [12,13]. Thus, the differences in cell signaling profiles could reflect contributions from genetic, epigenetic or environmental variables. Although samples were collected weekly, profiles from the same individual tended to cluster together, suggesting that kinomic profiles are stable (at least over a one-month period) (Figure 2a). Over this time frame, there would be considerable turnover of cells and kinases, offering some perspective on the imprinting of kinomic patterns within individuals. Several subjects displayed considerable changes in individual cell populations throughout the investigation; however, samples still clustered on the basis of individual, further supporting the existence of a stable kinotype.

### Individual-specific porcine kinome profiles

**Figure 3:**
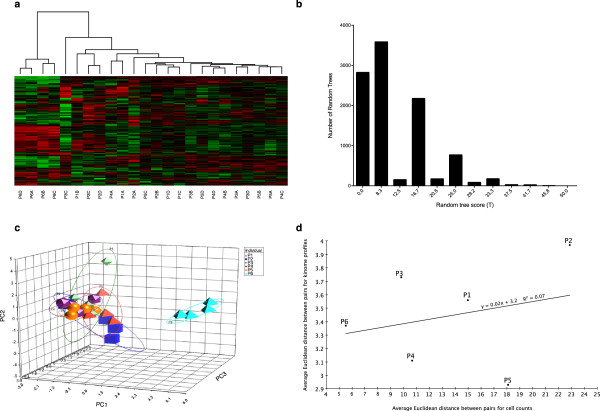
**Clustering of porcine kinome profiles. (a)** Hierarchical clustering of porcine kinome profiles. For details, see the caption for Figure 1a. **(b)** Distribution of random tree scores. For comparison, the score of the actual tree shown in part A is 50. **(c)** Three dimensional PCA. Individual subjects (P1, P2, P3, P4, P5, and P6) are color-coded. **(d)** Correlation between PBMC composition and kinome profiles. The procedure for generating this figure is the same as for Figure 2d.

Having demonstrated a stable, individual-specific kinome profile within human PBMCs, the concept of the kinotype was further challenged by considering the same cell population taken from pigs. In contrast to the diversity in the human subjects (gender, age, genetics, and lifestyle), the porcine subjects were littermates (siblings) housed within the same environment and sustained on the same diet. Remarkably, hierarchical clustering analysis still demonstrated a strong trend for the samples to cluster by individual (*T* = 50) (Figure 3a). The highest random tree score was also 50, which was achieved by a single tree (Figure 3b). The P-value was thus 0.0001, supporting the hypothesis that individual-specific kinotypes exist within porcine PBMCs. PCA analysis also demonstrated sample segregation on the basis of individual (Figure 3c). As with humans, there was no significant relationship between white blood cell population variance and kinome profile variance within individuals (Figure 3d).

### Species-specific differences in the kinotypes

Having demonstrated the existence of species-specific kinome profiles of human and porcine PBMCs, we sought to identify the phosphorylation events responsible for the species-specific clustering as well as to characterize the biological events associated with them. Given that the human and porcine samples clustered separately, one would expect many peptides to be differentially phosphorylated between the two species. This was indeed the case: 119 of the 297 peptides exhibited significantly increased phosphorylation in the human samples relative to the porcine samples, while 120 peptides exhibited significantly decreased phosphorylation. Because the sample size was large for each peptide (216 observations per species), statistical significance did not necessarily imply that the difference was large in magnitude: some of the peptides with small P-values also had small fold-change (FC) values. The P-values and FC values for all peptides can be found in Additional file 3.

**Figure 4:**
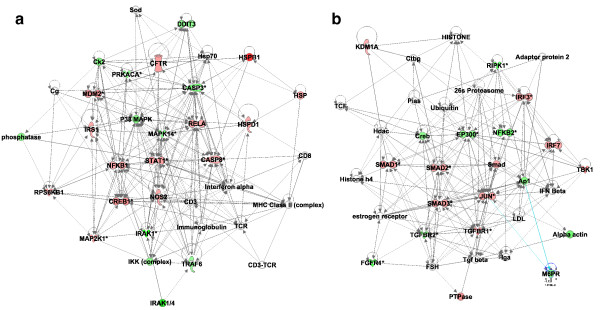
**Functional network analysis of differentially modulated kinome responses in humans as compared to pigs.** Peptide fold-change values for humans as compared to pigs were uploaded to IPA for functional network analysis. Differentially modulated functions were identified as follows: **(a)** cellular development, cell death and survival, cellular function and maintenance; and **(b)** gene expression, digestive system development and function, cell death and survival. As in the heatmaps, red indicates increased phosphorylation and green indicates decreased phosphorylation.

We have previously applied pathway over-representation analysis (ORA) to kinome data to infer cellular responses from the standpoint of signaling networks [23,24]. To provide initial biological insight into the observed species-specific kinotypes, here we used the Ingenuity Pathway Analysis software suite to perform functional network analysis, which provides information regarding the regulation of broad biological networks that can encompass multiple signaling pathways and cellular receptors. Differentially modulated functional networks identified from the comparison of the human and porcine profiles are presented in Figure 4. Functions related to cellular development, cell survival and death, and maintenance of cellular functions were over-represented, with phosphorylated mitogen-activated protein kinase (MAPK)-, signal transducer and activator of transcription (STAT)- and nuclear factor kappa-light-chain-enhancer of activated B cells (NFΚB)-regulated responses occupying central nodes of the functional network that exhibited the most significant change in modulation (Figure 4a). In addition, phosphorylated transforming growth factor (TGF)-β signaling pathway intermediates (including TGF-βRI and multiple SMAD proteins) formed central components of the functional network having the second-most significant change in modulation (Figure 4b). Additional biological verification and characterization of these kinotypic differences will be the subject of a subsequent study.

### Individual-specific differences in the kinotypes

The individual-specific kinome profiles observed in pigs and humans support the hypothesis that kinome profiling may provide a mechanism to identify biomarkers associated with particular traits. To determine which peptides were responsible for distinguishing the kinome profiles of the individuals of a given species, the standard deviation of the normalized intensity values (averaged over 4 samples per individual and 9 technical replicates per sample) among the 6 individuals was calculated for each peptide (Additional file 4). The standard deviations of the peptides varied greatly; in human, for instance, the most variable peptide (which corresponded to the protein HSP27) had a standard deviation of 0.56, whereas the least variable peptide (IKK-α) had a standard deviation of 0.04. The range was similar in pig, with the most variable peptide (IRAK4) having a standard deviation of 0.48 and the least variable peptide (iNOS) having a standard deviation of 0.02. A moderate correlation (*r* = 0.39) was found between the standard deviation of a given peptide’s response in human and the standard deviation of that peptide’s response in pig, suggesting that there is some commonality between the two species in terms of the variability of the response of a given peptide among different individuals.

## Discussion

Efforts to correlate phenotypes with biomolecular characteristics (e.g. nucleotide/amino acid sequences; patterns of expression/translation/modification) must often compromise between ease of technical application and biological relevance [25,26]. While static descriptors such as gene sequences are readily available, they often fail to capture the dynamic interplay between biological variables. In some situations, such as certain genetic disorders, the consequences associated with changes to a single biomolecule are sufficiently extreme to override this diversity. In other situations, interplay within the population of biomolecules may be of greater significance. These differences likely arise due to multiple levels of redundancy and plasticity that provide buffering for genetic differences, but also reflect individual responses. In these situations, it is most appropriate to define cellular responses at a level that reflects this interplay, ideally as close as possible to the phenotype. The challenge here is that unlike genetic polymorphisms, the levels of these biomolecules are dynamic and may be unique to particular tissues, cells or intracellular locations. From a practical perspective, there is also the need to be able to reliably quantify these biomolecules in a robust, cost-effective and high-throughput manner.

Protein kinases play a central role in regulating biological functions at the levels of proteins through to pathways and, ultimately, phenotypes. Changes in activities of individual kinases, through genetic defects or therapeutic modulation, can have profound impacts on the health and viability of an organism [27]. The growing interest in kinases in both basic and translational research has driven efforts to develop technologies that enable characterization of phosphorylation-mediated signal transduction [11,28]. Kinome analysis offers three key advantages over traditional gene and protein profiling: 1) individual kinase activities are often reliable indictors of phenotypic changes, 2) kinase profiling offers insight into cellular responses at the level of signalling pathways, and 3) as kinases are highly “druggable” [8,9], increased understanding of the biological role for kinases could aid therapeutic design and development.

To this end, our investigation sought to examine kinome responses in both inter- and intra-species comparatives. Our results demonstrate the existence of temporally stable species- and individual-specific kinotypes. Hierarchical clustering of the kinome data derived from human and porcine PBMCs showed that the kinome profiles clustered in a species-specific manner, suggesting that kinotype analysis could provide critical information regarding cell processes or signalling pathways that are differentially modulated across similar animal species.

In addition to verifying the existence of species-specific kinotypes, this finding may have further implications and applications. Recently, Seok and colleagues reported on the disparate correlation in genomic responses between human inflammatory diseases and murine inflammatory models [1]. This highlights a problem in basic and translational research where animal models of disease are largely validated through phenotypic similarity to human disease. Our results suggest that kinome analysis could be beneficial for the evaluation and assessment of animal models. Indeed, pigs are often employed as animal models of human disease. Thus, understanding the biological differences or predispositions between pigs and humans could inform situations where pigs may represent an appropriate animal model as well as influence the interpretation of emerging results. Further, our demonstration of species-specific kinotypes suggests that kinotype analysis could provide critical information regarding the degree of conservation of essential cell processes across animal species, in particular those that are closely related. Further, we postulate that kinome analysis could provide information regarding conserved mechanisms of molecular pathogenesis between humans and animals routinely used in models of human malignancies. With respect to the current US Food and Drug Administration Animal Efficacy Rule [29,30], kinome analysis could provide insight in investigations for which human efficacy trials are neither feasible nor ethical and, in particular, in the selection of animal models that best recapitulate human molecular disease.

From the standpoint of drug development, the analysis of individual-specific kinotypes could help define the temporal stability of particular drug targets as well as their conservation across the population. Personalized medicine is based on an appreciation that natural biological variation exists within outbred populations. Customizing diagnoses and therapies to an individual rather than an assumed biological norm has the potential to maximize treatment efficacy while minimizing side effects [31,32]. The implementation of personalized medicine at a molecular level depends on the identification of biomarkers that accurately predict some aspect of disease, such as onset, prognosis or treatment efficacy. It is our belief that kinotype analysis could facilitate this process.

Beyond this study, there is a substantial opportunity for future work in terms of expanding the number of model organisms considered. Perhaps the most important information that could be derived from an analysis involving several species would be to determine which has a kinome profile most similar to that of human. More generally, it would be interesting to compare the clustering of the different species’ kinome profiles with those obtained from traditional sequence-based phylogenetic approaches (e.g. mitochondrial 16S rRNA gene comparisons). Answering the question, “Is there a strong relationship between genetic similarity and kinotypic similarity?” would be hugely beneficial in terms of selecting appropriate animal models and understanding how well the responses of a given model might reflect those in human. Another avenue for future work derives from the fact that the number of samples could become quite large if several species are considered, especially if many individuals are tested per species and/or many samples are taken per individual. In this study, four samples were taken from each of six individuals from each of two species, for a total of 48 samples. In addition, each sample was exposed to a peptide array with nine intra-array technical replicates per peptide sequence. Kinome microarrays are relatively inexpensive; nonetheless, in order to provide accurate comparisons while simultaneously minimizing costs, it could be beneficial to characterize the number of intra-array replicates per sample, samples per individual, and individuals per species required to accurately reflect the level of kinotypic similarity among species and individuals.

## Conclusions

The identification of phosphorylation signatures associated with disease states using kinome analysis may become an important tool in basic and translational research. This study suggests that these signatures must be considered in the context of the range of variability at the level of both species and individual. For instance, if an animal is being considered as a model for a particular disease—and in particular, if host responses are being evaluated at the kinome level—then species-specific baseline levels of kinase activity may need to be taken into account. The same concept applies in the context of personalized medicine: a treatment that is effective in some individuals may not be effective in other individuals, and it is possible that an individual’s kinome profile may be predictive of the efficacy of a given treatment. Of course, further studies are needed to precisely define the methodology needed for incorporating kinome analysis into both treatment studies and studies involving animal models. While considering baseline kinomic responses may prove complicated, the discovery of complex biomarkers, in particular those associated with kinase activities, has tremendous potential to inform research involving animal models as well as personalized medicine.

## Methods

### PBMC isolations

Human and porcine blood samples were collected weekly for 4 consecutive weeks. For humans, 6 unrelated individuals (3 male and 3 female) diverse in age (21–55), race, diet, and health status were selected. Porcine samples were obtained from 6 littermates (3 male and 3 female) beginning at 4 weeks of age. Pigs were housed within the same pen and fed the same diet. Bleeds were performed at the same time each day to minimize variability associated with circadian rhythms and postprandial effects. PBMCs were isolated as previously described [33]. Aliquots of 1 × 10^7^ PBMCs were snap-frozen in liquid N_2_ and stored at -80°C for kinome analysis. All animal procedures were performed in accordance with the standards of the Canadian Council on Animal Care. Human subjects provided written informed consent before participation. This procedure, and all research done using these samples, was done in accordance with the University of Saskatchewan Clinical Research Ethics Board.

### Peptide arrays

Design, construction and application of the peptide arrays were based on previous protocols with modifications [34]. A commercial provider, JPT Peptide Technologies (http://www.jpt.com), was contracted to fabricate the arrays. Peptides from proteins representing a wide variety of signalling pathways were included on the arrays. Specifically, 297 peptide sequences were chosen, each of which was spotted 9 times on the same array (i.e. there were 9 intra-array technical replicates per peptide). It should be noted that this type of technical replicate is distinct from inter-array technical replicates, for which the entire process (from incubation of the sample with the array to scanning the array using image analysis software) is repeated multiple times. The technical replicates for a given peptide were averaged prior to performing clustering analysis. The exact composition of the array, including spot coordinates, block layouts, and peptide sequences, is given in Additional file 5.

All peptides on the array are found as exact matches in both the human proteome and the porcine proteome; as such, the same arrays were used for both species, enabling a direct comparison of all kinome profiles. Kinome array experiments for both species were performed on the same day to minimize technical variance and performed as described previously [35]. Each resulting dataset contained the signal intensities associated with all 9 replicates of the 297 peptides from a given individual at a given time point. All data processing and analysis was done using the Platform for Intelligent, Integrated Kinome Analysis (PIIKA) software [36], which is freely available for non-commercial use at http://saphire.usask.ca/saphire/piika.

### Evidence for individual kinotypes in humans and pigs

To determine whether unique kinotypes existed within individuals, the following statistical question was addressed: “Do samples from the same individual cluster more closely than expected by chance?” Samples from the 6 individual humans and pigs were separately subjected to hierarchical clustering using (1 – Pearson correlation) as the distance metric and McQuitty linkage as the linkage method. We defined a metric describing how close to perfect (i.e. all samples from the same individual clustering together) the actual clustering was. As each iteration of the hierarchical clustering algorithm results in a bifurcation, the resulting dendrogram can be represented as a binary tree wherein each leaf represents one of the 24 samples, and each internal node represents a cluster of 2 or more samples. For each individual *i*, a score *s*_*i*_ was computed. If some internal node in the binary tree had, as descendants, four leaves corresponding to individual *i* and none corresponding to any other individual, then *s*_*i*_ = 4. If the same criteria could be satisfied but with only 3 leaves corresponding to individual *i*, then *s*_i_ = 3, and similarly for *s*_*i*_ = 2 and *s*_*i*_ = 1. If there were no internal nodes having, as descendants, only leaves corresponding to individual *i*, then *s*_i_ = 0.

The score for the entire tree was $S=\sum_{i=1}^{6}s_{1}$, with the maximum possible score being 24. This was then expressed as a score out of 100: *T = S /* 24 × 100. To determine whether *T* was greater than would be expected by chance, an empirical statistical distribution was derived by generating 10,000 random trees. Each tree was created by randomly rearranging the normalized intensity values for the peptides within a given sample. The average normalized intensity value (over the 9 technical replicates) for a given peptide X was randomly assigned to a different peptide Y from the same sample, and this was done for all peptides across all samples. For each random tree *j,* hierarchical clustering was performed and a score *T*_*j*_ was calculated as described above. The P-value for a given score *T* was then calculated as the proportion of scores *T*_*j*_ that were ≥ *T*.

### Evidence for species-specific kinotypes

To answer the question, “Do samples from the same species cluster more closely than expected by chance?”, hierarchical clustering was performed with all samples from both species at once. The same scoring metric as above was used, but with only 2 “individuals” – human and pig, each with 24 samples. Thus, $S=\sum_{i=1}^{2}s_{1}$, with *s*_*1*_ and *s*_*2*_ denoting the scores for the human and porcine samples, respectively, and *T* = *S* / 48 × 100. Statistical significance was determined as above.

### Correlating cell composition and kinome profiles

Blood contains a dynamic population of cells that, based on their unique functions, likely exhibit distinct signalling activity. Thus, species- or individual-specific kinome patterns could reflect unique blood cell populations. To account for this potential variability, differential counts were performed on each sample. Importantly, kinome analysis was performed solely on PBMCs (lymphocytes and monocytes) and excluded polymononuclear cells (PMNs). We investigated whether there was any correlation between kinome profiles and relative abundance of PBMCs as follows.

The level of variability within an individual’s kinome profile over time was determined by finding the Euclidean distance between each of the 6 possible pairs of samples for the same individual (week 1 and week 2, week 1 and week 3, etc.) with respect to the average normalized intensities for each peptide. Specifically, each sample was represented as a vector of length 297, where each element represented the average normalized intensity value for a peptide on the array. For a given pair of samples *x* and *y*, the Euclidean distance was calculated as $\sqrt{{\sum_{i=1}^{297}\left(x_{i}-y_{i}\right)}^{2}}$. The level of variability in a given individual’s kinome profile was the average of all 6 Euclidean distances. The level of variability in cell counts over time was assessed similarly, except the values of a given vector represented counts for a given cell type. As such, these vectors were of length 2 (lymphocytes and monocytes). To determine whether there was a relationship between the variables mentioned above, a scatterplot was created for each species wherein the independent axis represented variability in cell counts for a given individual, and the dependent axis represented variability in kinome profile. Linear regression was performed, and the coefficients of the regression line and the *R*^*2*^ value were calculated for each species.

### Species-specific differences in the kinotypes

Statistical tests for identifying peptides differentially phosphorylated in the human samples compared to the porcine samples were carried out as described previously [36]. Specifically, for each peptide, a t-test was done by comparing all 216 human observations (6 subjects × 4 samples per subject × 9 technical replicates per sample) against all 216 porcine observations. A peptide was considered to be differentially phosphorylated if the resulting P-value was less than 0.05. Pathway overrepresentation analysis was performed as previously described [36], except that the Ingenuity Pathway Analysis software suite was used instead of InnateDB.

### Individual-specific differences in the kinotypes

In order to identify peptides driving the differences between the kinome profiles of different individuals, the 36 normalized intensity values for a given individual (4 samples per individual × 9 technical replicates per sample) were averaged for each peptide. Within each species, the standard deviation of these values for the 6 individuals was calculated. Peptides with high standard deviation had the greatest variation in responses among the individuals, while peptides with low standard deviation had the most consistent responses.

## Competing interests

The authors declare that they have no competing interests.

## Authors’ contributions

SN and PG conceived the study. ES performed the peptide array experiments. BT performed the majority of the data analysis, with additional contributions by JK, AK, and SN. BT, JK, AK, PG, and SN interpreted the data. BT, JK, and SN wrote the paper, with substantial revisions by AK. All authors read and approved the final manuscript.

## Supplementary Material

Additional file 1 **Raw array intensity data for all human and porcine samples.** Columns correspond to samples and rows correspond to peptides. The rows are in groups of 9, representing the values for the 9 technical replicates associated with a given peptide. The first column for a given sample represents the foreground intensity, while the second column represents the background intensity. Click here for file

Additional file 2 **Intensity data after background subtraction and normalization.** Columns correspond to samples and rows correspond to peptides. Unlike Additional file 1, there is only one row corresponding to a given peptide; however, there are 9 columns for each array, which give the normalized intensity values for the 9 technical replicates for that peptide. Click here for file

Additional file 3 **Comparison of human and porcine kinome responses.** The fold-change value between human and pig is given for each peptide on the array, along with P-values for increased and decreased phosphorylation. Click here for file

Additional file 4 **Inter-individual variability of peptide responses.** The left-hand block of cells contain, for a given peptide and individual, the mean normalized intensity value among the 36 observations (4 samples per individual and 9 technical replicates per sample). Column O contains the standard deviation of the 6 human means, while column R contains the standard deviation of the 6 porcine means. Click here for file

Additional file 5 **Composition of the peptide arrays.** This GenePix Array List (GAL) file contains the exact composition of the peptide array used in this study, including the location of each spot and the peptide contained in that spot. It is in plain-text format and can thus be read by any text editor. Click here for file
